# Membrane Technologies in Wastewater Treatment: A Review

**DOI:** 10.3390/membranes10050089

**Published:** 2020-04-30

**Authors:** Elorm Obotey Ezugbe, Sudesh Rathilal

**Affiliations:** Department of Chemical Engineering, Faculty of Engineering and the Built Environment, Durban University of Technology, Durban 4000, South Africa; rathilals@dut.ac.za

**Keywords:** membrane technology, wastewater, potable water, fouling

## Abstract

In the face of water shortages, the world seeks to explore all available options in reducing the over exploitation of limited freshwater resources. One of the surest available water resources is wastewater. As the population grows, industrial, agricultural, and domestic activities increase accordingly in order to cater for the voluminous needs of man. These activities produce large volumes of wastewater from which water can be reclaimed to serve many purposes. Over the years, conventional wastewater treatment processes have succeeded to some extent in treating effluents for discharge purposes. However, improvements in wastewater treatment processes are necessary in order to make treated wastewater re-usable for industrial, agricultural, and domestic purposes. Membrane technology has emerged as a favorite choice for reclaiming water from different wastewater streams for re-use. This review looks at the trending membrane technologies in wastewater treatment, their advantages and disadvantages. It also discusses membrane fouling, membrane cleaning, and membrane modules. Finally, recommendations for future research pertaining to the application of membrane technology in wastewater treatment are made.

## 1. Introduction

All activities of mankind are water dependent. With the increase in human population, tons and tons of wastewater are produced everyday across the domestic, industrial, and agricultural sectors. Freshwater resources, however, do not get replenished to accommodate the ever-increasing population and its water usage needs. This has led to intense competition and unfair distribution of the limited freshwater resources among the various sectors. Consequently, many people around the world, especially in developing countries, lack access to potable water. Again, agricultural activities are heavily affected, as farms lack access to enough water resources for all year-round irrigation and livestock production. The evidence of these situations is seen across the world, especially in the Middle East, Africa, Asia, and Latin America. The facts are glaring, such as 2.1 billion people living without safe drinking water at home, and nearly four billion people experience severe water scarcity during at least one month of the year [1,2].

Wastewater generation is inevitable as it forms an integral part of the value chain in all sectors of life. In the oil refinery industry, every one barrel of crude oil processed generates about 10 barrels of wastewater [3]. In an infrastructure report by the South African Institution of Civil Engineers, titled *SAICE Infrastructure Report Card for South Africa, 2011,* it was noted that an average of 7589 mega liters per day of wastewater is transported across South Africa [4]. All these wastewaters are clean water with contaminants. With efficient wastewater treatment, freshwater resources can be supplemented, and potable water can be made accessible to all. This seems to be the most obvious way of dealing with water scarcity [5].

In this vein, several efforts have been made over the years to introduce various wastewater treatment technologies such as conventional filtration, coagulation-flocculation, and biological treatment systems among others. There is also improvement of already existing technologies to meet current discharge or reuse standards. One of the wastewater treatment technologies that have seen a major boost over this period is membrane technology. Membrane technology has grown significantly in the last couple of decades due to the benefits it offers in water and wastewater treatment. With significant reduction in the size of equipment, energy requirement and low capital cost, membrane technology offers many prospects in wastewater treatment [6]. According to Singh and Hankins [7], membrane technology has the potential of bridging the economical and sustainability gap, amid possibilities of low or no chemical usage, environmental friendliness and easy accessibility to many. That is, membrane technology has proven to be a more favorable option in wastewater treatment processes in recent times.

Even though membrane technology is not a new invention, the varying nature and complexity of wastewater makes room for more improvements, in terms of efficiency, space requirements, energy, quality of permeate, and technical skills requirements. Again, there is continuous modification of membrane modules and membrane elements to enhance the reduction in membrane fouling, which is a major challenge for membrane processes. The possibility of combining two or more membrane processes with each other, or with other forms of technology like coagulation or adsorption, in a hybrid fashion is also continuously being explored, developed and applied in many wastewater treatment facilities [7,8,9].

This paper reviews the application of membrane technology in wastewater treatment. It considers the advantages and the disadvantages of these processes. Again, the paper touched on some general terms like membrane modules and their applications, concentration polarization, membrane fouling, and membrane cleaning techniques. It also discusses the prospects of membrane technology.

## 2. Membrane Technology for Wastewater Treatment

Basically, a membrane is a barrier which separates two phases from each other by restricting movement of components through it in a selective style [10]. Membranes have been in existence since the 18^th^ century. Since then, a lot of improvements have taken place to make membranes better suited for many different applications [11].

Characteristically, membranes can be classified as isotropic or anisotropic. Isotropic membranes are uniform in composition and physical structure. They can be microporous; in which case their permeation fluxes are relatively high compared to when they are nonporous (dense) where their application is highly limited due to low permeation fluxes. Isotropic microporous membranes are widely applied in microfiltration membranes. Anisotropic membranes on the other hand are non-uniform over the membrane area and are made up of different layers with different structures and composition. These membranes have a thin selective layer supported by a thicker and highly permeable layer. They are particularly applied in reverse osmosis (RO) processes [12,13].

In terms of membrane material make up, membranes are classified as either organic or inorganic. Organic membranes are made from synthetic organic polymers. Mostly, membranes for pressure driven separation processes (microfiltration, ultrafiltration, nano filtration and reverse osmosis) are made from synthetic organic polymers. These include polyethylene (PE), polytetrafluorethylene (PTFE), polypropylene, and cellulose acetate among others [14]. Inorganic membranes are made from such materials as ceramics, metals, zeolites, or silica. They are chemically and thermally stable and used widely in industrial applications like hydrogen separation, ultrafiltration, and microfiltration [13,15].

**Figure 1:**
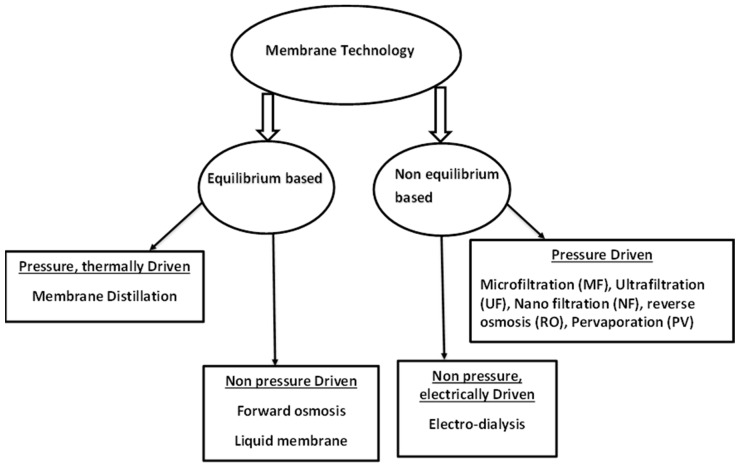
Schematic representation of some membrane processes. Modified from [16].

Movement of media through the membranes is based on different driving forces. There are equilibrium based membrane processes, non-equilibrium based membrane processes, pressure driven and non-pressure driven processes [16]. The schematic diagram below (Figure 1) shows a summary of some of these techniques according to their driving forces. These membrane techniques are discussed individually below.

### 2.1. Pressure Driven Membrane Processes

Pressure driven membrane processes are by far the most widely applied membrane processes in wastewater treatment, from pretreatment to post-treatment of wastewater. These processes rely on hydraulic pressure to achieve separation. There are four main types of these processes. These are microfiltration (MF), ultrafiltration (UF), nano filtration (NF), and reverse osmosis (RO). The main difference exhibited by these processes, apart from their pressure requirements, is their membrane pore sizes [17]. Table 1 provides a summary of the main features of these processes.

Among the pressure driven membrane processes, RO is highly known for its efficiency in separating small particles including bacteria and monovalent ions like sodium ions and chloride ions up to 99.5% [18]. RO has been at the forefront of water reclamation through wastewater treatment and desalination of seawater for a long time. During reverse osmosis, a hydrostatic pressure is generated that is strong enough to overcome the intrinsic osmotic pressure of the feed. This is against the natural osmosis process. For the complete process, water molecules are absorbed onto the membrane surface (under pressure). These molecules diffuse through the membrane material and finally desorb at the permeate side of the membrane for collection [19].

Different combinations of these pressure driven membrane processes have been applied in different wastewater treatment settings. In some cases, they serve as pre-treatment to other unit processes. In an experiment, Nataraj, et al. [20] combined NF and RO to treat distillery wastewater in which an average of 98% of contaminants (colour, total dissolved solids, chemical oxygen demand, and potassium) were removed successfully. In another application, UF and RO were combined in a pilot scale plant to treat wastewater from reactive dye printing. After the UF, the permeate still fell short of the discharge limits, however the RO permeate was fit for discharge and reuse. Contaminants such as urea, sodium alginate, reactive dye and oxidizing agents were successfully removed [21]. Several other instances of applying these pressure-driven membrane processes are shown in Table 2.

As seen in most of the applications listed above, MF, UF, and NF usually serve as pretreatment steps to RO. This is to reduce fouling of the RO membrane and to enhance the maintenance of constant flux. This also serves as a multi-barrier treatment for removal of contaminants from wastewater [32,33]. Pressure driven membrane processes have undoubtedly made water reclamation from wastewater a good option. However, the challenge still remains with the energy requirements due to the pressure.

### 2.2. Forward Osmosis (FO)

**Figure 2:**
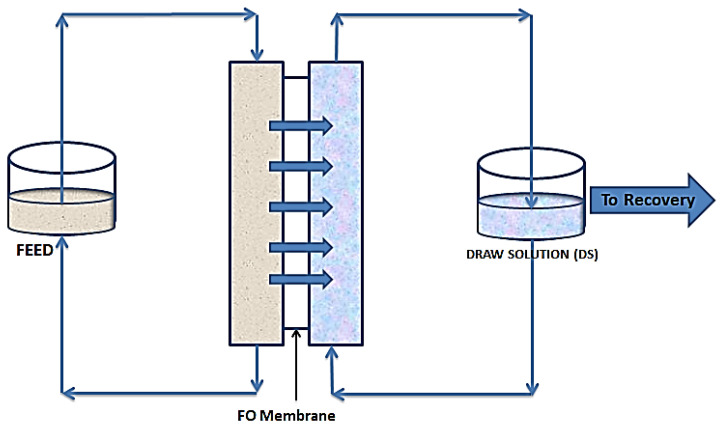
Schematic diagram of forward osmosis.

As shown in Figure 2, FO follows the natural osmosis process where water molecules are drawn from one solution to the other, through a semipermeable membrane. In this case a draw solution (DS), which is highly concentrated, is used to provide a concentration gradient to draw water molecules from the feed solution (FS). This gradient provides the needed osmotic pressure difference to drive water molecules from the FS to the DS. This movement continues till an equilibrium of chemical potential is reached [34]. Unless for niche applications, where the water being drawn from the feed forms part of the product, there is always the need for a recovery unit. This unit simultaneously recovers fresh water and regenerates the draw solution [35].

FO has been applied for the treatment and concentration of different streams of wastewater. Holloway, et al. [36] applied FO in concentrating anaerobic digester centrate in which an RO system was used to recover and reconstitute the draw solution. Similarly, York, et al. [37] applied FO in a landfill leachate management using NaCl as a draw solute and RO to recover and reconstitute the draw solution. Up to 95% of the permeate was recovered. In other applications, Zhang, et al. [38] combined FO and MD to recover water from oily wastewater. Haupt and Lerch [39] conducted a series of FO experiments to investigate the applicability of FO in an automobile production site and dairy industry. Five different wastewater streams were utilized in turn, as DS and as FS. These wastewater streams were; wastewater treatment RO concentrate, cheese brine, cathodic dip painting rinsing water, paint shop pre-treatment wastewater and cooling water circulation water. Where these effluents were used as FS, 1 mol/L NaCl solution was used as DS. When they were used as DS, deionized water was used as FS. There was also the combination of two or more wastewaters for use as DS or FS. It was found out that cooling tower circulation water and cathodic dip painting rinsing water were very unsuitable for use as DS and therefore cannot be applied in FO in an automobile production site. It was however found out that cathodic dip painting rinsing water and paint shop pre-treatment wastewater proved well as FS when 1 mol/L NaCl was used as DS and can therefore be utilized in an automobile production site in wastewater treatment. The cheese brine was found to be promising for use as DS in dairy wastewater treatment. Thus, FO was found to be applicable in the treatment of these wastewaters. Other applications of FO in wastewater treatment are shown in Table 3.

In their write-up on “Forward Osmosis for Sustainable Water Treatment”, Shen, et al. [46] noted that the method for fresh water recovery after FO is greatly dependent on the kind of draw solute used. Where monovalent ions such as sodium and chloride form part of the draw solution, RO is mostly employed for the recovery whereas multivalent ions, hydrophilic nanoparticles, micelles and polyelectrolytes would require membranes with larger pore sizes like ultra-filtration or nano filtration. FO has several advantages. The process does not require external pressure (especially for niche applications of FO), which makes energy consumption lower compared to pressure driven processes. Fouling reversal and water cleaning is also easier due to the use of osmotic pressure for separation. Flexibility in choosing draw solution makes it easy to customize products, either for freshwater recovery or for other purposes like pharmaceuticals and beverage production in which case properties of products are maintained, since no pressure or heat is applied. Furthermore, the regeneration and reuse of DS is advantageous in saving cost. Challenging (highly concentrated) FS are better treated with FO. For example, for a highly saline feed, more energy would be required by RO to overcome the osmotic pressure, hence making the choice of FO a better one [46,47,48,49].

With all these promising features of FO, it has some drawbacks that require attention. Apart from niche applications of draw solution, where the draw solute forms part of the final product, further separation is needed to recover fresh water. Low permeate flux due to concentration polarization (CP) is another drawback with FO. This CP affects the net osmotic pressure, hence reducing permeate flux. Again, energy requirements for FO increases with decreasing molecular weight cut off (MWCO). This is because regeneration of draw solutes would require membranes with smaller pores and more pressure like RO. This in effect increases the overall energy requirements [46,47,48,49].

**Draw Solution Selection and Recovery for FO System**: As afore-mentioned, FO systems depend on concentration gradients to cause the movement of water molecules. This concentration gradient is provided by the draw solution (DS). Draw solutions are formed when draw agents or solutes are homogeneously dissolved in water to form solution [50]. Draw solutions play a significant role in FO processes, as they influence permeation flux and cost of regeneration [51]. Many draw solutions exist. Typical properties of draw solutions include the following; they are characterized by high osmotic pressure, which is their most important feature. Again, DS should have low reverse solute diffusion to FS and should be easily regenerated [49]. It is also important that DS is non-toxic, highly stable and highly soluble in water to avoid precipitation [52]. Generally, draw solutes come in different forms viz organic (sucrose, glucose, fructose, EDTA, sodium polyacrylate, sodium lignin sulfonate (NaLS), etc.), inorganic (NaCl, MgCl_2_, Na_2_SO_4_, KCl, KNO_3_, etc.), magnetite nano particles (Fe_2_O_4_), gases and volatile compounds (ammonia and CO_2_), [53,54]. The kind of draw solute recovery method to employ depends on the nature of the draw solute used. In general, membrane separation (RO, NF, UF, MD) processes are preferred for salt-based draw solute recovery. For gases and volatile compounds such as SO_2_, NH_3_/CO_2_ thermal separation is used. Other methods include precipitation for sulphate base draw solutes like Al_2_(SO_4_)_3_, Mg(SO_4_), Cu(SO_4_) and stimuli based recovery process for hydrogels and magnetite nano particles [55,56]

### 2.3. Electro-Dialysis (ED) and Electro-Dialysis Reversal (EDR)

**Figure 3:**
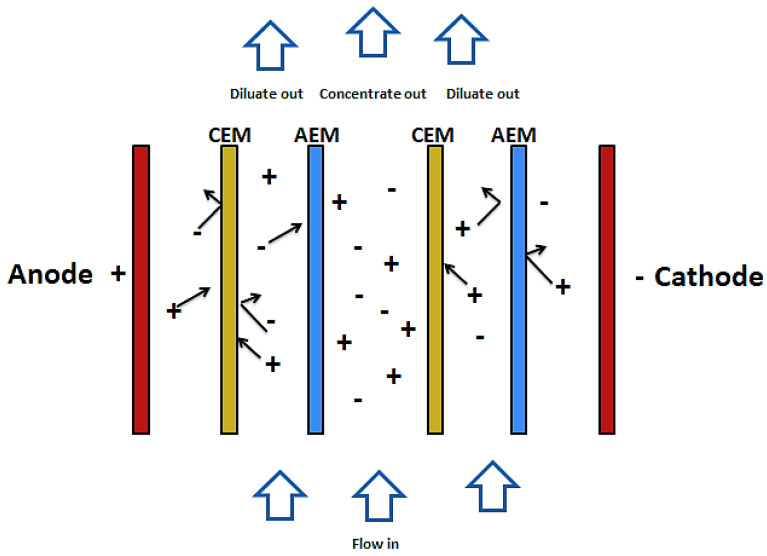
Schematic diagram of ED.

Electro-dialysis and electro-dialysis reversal are processes that combine electricity and ion-permeable membranes to separate dissolved ions from water. These processes make use of an electric potential to transfer the ions from a dilute solution to a concentrated solution through an ion-permeable membrane [57]. As shown in Figure 3, during the electro dialysis, two types of ion exchange membrane are used. One is permeable to anions and rejects cations and the other is permeable to cations and rejects anions. There are also two streams of solutions. The concentrate and the diluate (feed). When electric current is passed through the system, ions from the diluate migrate into the concentrate through oppositely charged membranes (cations migrate to the cathode whiles anions migrate to the anode). The cations are then retained by the positively charged anion-exchange membrane (AEM). Likewise, the anions are retained by the cation-exchange membrane (CEM). The outcome of this is a feed stream depleted of ions while the concentrate stream becomes rich in ions [58,59].

EDR involves the periodic reversal of the electrodes of the membrane and hence reversing the movement of ions. This causes concentrated streams to be diluted and diluted streams to be concentrated, hence reducing fouling of the membrane [60]. ED and EDR have been applied in many ways in wastewater treatment. Table 4 shows some common applications.

ED and EDR are very useful in wastewater treatment mainly to remove total dissolved solids (TDS) and other ionized constituent particles. ED and EDR have very high-water recovery rate and require little pretreatment for feed water. There is also less membrane fouling due to process reversal and the technology can be combined with renewable energy sources [67].

However, ED is not suitable for wastewater streams with high salinities because desalination energy is proportional to the ions that are removed. This would then be very expensive to operate. The process also does not remove non-ionized compounds and substances such as viruses and bacteria, which are very harmful. This implies that a post treatment would be required, which would make the process expensive. Furthermore, due to the generation of chlorine gas at the anode, corrosion can set in [7,57,58,59].

### 2.4. Pervaporation

**Figure 4:**
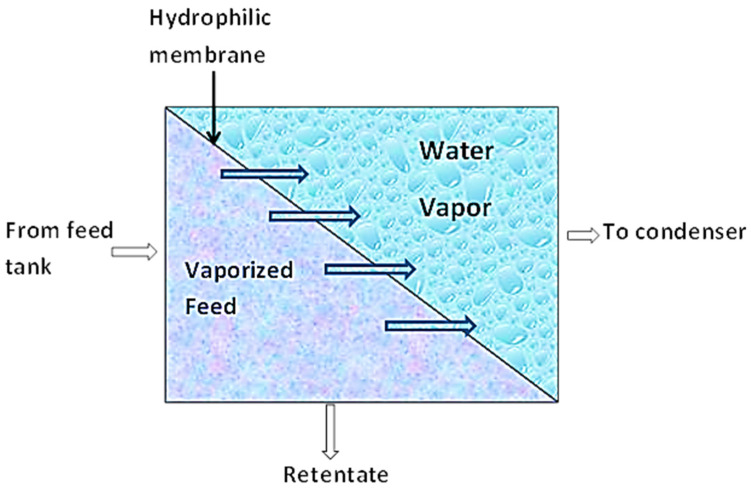
Schematic diagram of Pervaporation.

This separation technique combines membrane permeation and evaporation to separate liquid mixtures based on a preference. As shown in Figure 4, the liquid mixture is fed to the membrane on the one side while the permeate evaporates on the other side [68]. During this process, sorption of the permeate in the upstream occurs. By this, the more permeable component of the liquid mixture is sorbed onto the membrane (nonporous polymeric membrane or molecularly porous inorganic membrane). These components then diffuse through the membrane under the influence of a concentration gradient of the diffusing species and subsequently evaporate at the downstream phase of the membrane. The vapor is then condensed and recovered as liquid. This mode of mass transport across the membrane is known as the solution–diffusion model [68,69].

This technology has been applied mainly in ethanol-water separation. It is, however, being explored for wastewater treatment in many areas of production.

Edgar, et al. [70] applied pervaporation in micro irrigation of plants from wastewater. In the experiment, a dense hydrophilic pervaporation membrane was placed at vantage positions in the soil. Synthetic wastewater in a feed tank was circulated over the membranes where the permeate flux and enrichment of the wastewater (contaminant rejection) were monitored. The results showed that this technology has prospects in the treatment of brackish ground water or wastewater for micro irrigation purposes.

In a pilot scale experiment to remove organic solvents (benzene, toluene, naphtha, butane, ethyl ether, etc.) from dilute aqueous streams, Wijmans, et al. [71] used 100 organophilic membranes to remove and concentrate these solvents from the aqueous stream. It was observed that it was possible to concentrate the organic solvents at least 50–100-fold, thereby making available a cleaner stream of wastewater for re-use or discharge.

In a similar work, Kondo and Sato [72] used a polyether block amide (PEBA) membrane, which is aromatic hydrocarbon selective to remove phenol from industrial wastewater discharged from a phenolic resin process. The wastewater contained up to 10% phenol and other contaminants. After experiments, phenol concentrations detected were below 300 mg/L. the characteristic nature of pervaporation makes it applicable for target specific contaminants. Table 5 shows some target specific applications of pervaporation in wastewater treatment.

Due to the specialty in application, pervaporation membranes are specially designed to have higher affinity for the component to be separated. This implies that the chemical nature and structure of the membrane plays a significant role in achieving the intended separation [79]. Other factors that affect the processes of pervaporation include feed concentration, partial pressure, temperature and feed flow rate [80].

In addition to its ability to separate liquid mixtures where conventional separation processes are limited, pervaporation is known to be an energy saving and eco-friendly technology [81]. There are, however, some drawbacks with this technology. Large industrial application is still not reached due to its highly sensitive operating conditions. Again, the application of pervaporation beyond dehydration is on the low side due to a lack of specialized membrane and the cost of these membranes [68,82].

## 3. Hybrid Membrane Processes

Hybrid membrane processes refer to the combination of one or more membrane techniques with other unit processes like coagulation, ion exchange, adsorption or other membrane processes to give a better performance than either of the technologies as a standalone process [83]. Each component within the hybrid process tends to complement the drawbacks of the other, thereby enhancing the production of more quality treated water [84]. With an increase in stringent discharge standards and the radical search for alternative sources of water, more hybrid membrane processes are being explored. Also, due to the high risk of fouling of membranes, other unit processes such as coagulation, flocculation, sedimentation, or other membrane processes are introduced as pretreatment steps to reduce fouling of the membranes [8].

The main focus for hybrid membrane processes is to produce water for drinking or with different degrees of quality for other applications like irrigation, janitorial services or to use as cooling water in industrial processes. In a series of applications, low pressure membrane processes (MF, UF) were combined with activated carbon in the treatment of contaminated ground water for drinking. The combination was able to remove particulate matter and protozoa including Giardia, pathogenic bacteria, Cryptosporidium and dissolved organic matter, making potable water available for use [8]. In a similar fashion, Xiangli, et al. [85] combined coagulation as a pre-treatment to UF, in which 10,000 m^3^ per day of potable water was produced from a highly turbid river, the Taihu river, China. The combination ably reduced fouling of the UF membrane, leading to the maintenance of specific flux of up to 190,200 L/m^2^·hr·bar.

### 3.1. Forward Osmosis—Reverse Osmosis Hybrid Systems

**Figure 5:**
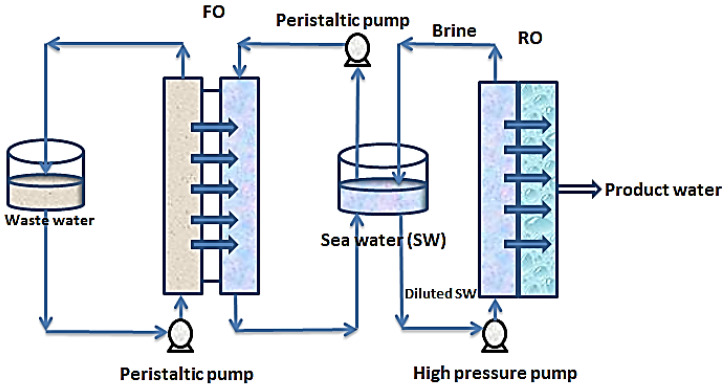
Hybrid FO-RO system for simultaneous seawater desalination and wastewater treatment.

In other related developments, FO-RO hybrid systems are being explored for simultaneous treatment of wastewater and desalination of seawater. Figure 5 shows a simplified set up of an FO-RO set up for simultaneous wastewater treatment and seawater desalination.

In this setup, wastewater of relatively low salinity is used to dilute seawater to reduce the pressure required by reverse osmosis for desalination. In this process, the chemical potential of the seawater provides a concentration gradient which causes diffusion of water from the wastewater stream through a semi-permeable membrane into the seawater stream [86]. As shown in Figure 5, the wastewater stream and the saline stream are circulated over the opposite sides of a semi-permeable membrane (designated FO). Through the contacts made with the membrane, water molecules move from the wastewater stream into the seawater stream thereby diluting the seawater stream before the reverse osmosis step. The brine from the RO is recycled into the seawater feed tank to boost the concentration gradient. Consequently, the wastewater is treated simultaneously with the desalination of the seawater. This hybrid process has a huge advantage of low external energy requirements for solvent-solute separation and serves as a multi-barrier for contaminant removal [87].

### 3.2. Membrane Bioreactors

**Figure 6:**
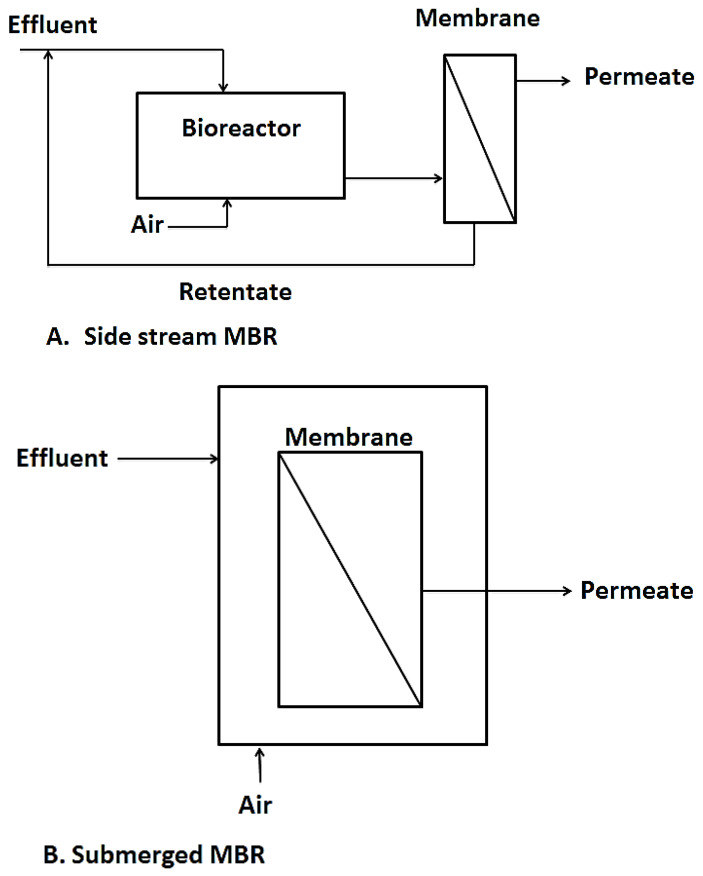
The two types of MBR, diagram taken from [92].

Another interesting hybrid membrane process worth talking about is membrane bioreactors. This is a technology that combines biological processes like activated sludge and membrane processes like UF, NF, MF, etc. for wastewater treatment purposes or for resource recovery from wastewater [7,88]. Over the past couple of decades, MBRs have surfaced as efficient wastewater treatment techniques as they fill in the gaps left by conventional activated sludge processes such as their inability to cope with fluctuations in effluent flow rates and composition as well as their failure to meet higher effluent discharge limits for reuse purposes. MBRs also save much space compared to conventional treatment systems [89,90]. Two configurations are currently in use, namely side stream MBR and immersed MBR as shown in Figure 6. The side stream MBR was the first to be introduced. With the side stream MBR, the membranes or filtration element are installed outside the bioreactor, needing an intermediate pumping system which transfers the biomass for filtration and residue from the filtration set up back to the bioreactor. This set up is advantageous, in that the membrane module is easily accessible for cleaning, however, due to the high energy and pressure requirements, the side stream MBRs have had limited application in recent years [91].

In the immersed or submerged MBR, the membranes are directly immersed in the tank containing the biological sludge and the treated permeate is extracted as shown in Figure 5B. This configuration was first introduced by Yamamoto, et al. [93]. It became highly patronized due to its simplicity and low energy requirements compared to the side stream MBR. It however has its own drawbacks in difficulty in cleaning the membrane units as the units are immersed in the bioreactor [94,95].

### 3.3. Membrane Distillation

Membrane distillation (MD) is a growing membrane technology being explored. This hybrid membrane process is said to have existed for more than 50years, with little development for large scale or commercial use [7]. Membrane distillation can be defined as the application of heat to separate substances based on their volatilities. In this technique, water vapor is transported across a hydrophobic microporous membrane, based on vapor pressure gradient across the membrane [48,96]. This heat driven process is mainly beneficial for separating feed solutions with high water content. MD is adapted to use low grade thermal energy (<100 °C) to provide the needed vapor pressure difference between the feed side and the product side of the membrane [48,97,98]. For example, Alkhudhiri, et al. [99] applied MD in the treatment of produced water from the Arabian gulf where they found MD very promising in terms of achieving high permeate flux and energy utilization. Kiai, et al. [100] applied MD in the treatment of table olive wastewater high in phenols. Membranes of different pore sizes were used to evaluate the effects on permeate quality and phenol concentration. Product quality was found to be high with phenolic concentration below 16 mg of TYE/L (tyrosol equivalent per liter) and conductivity levels of less than 193 µS/cm. In textile wastewater treatment, Calabrò, et al. [101] studied the energy efficiency of a MD system with respect to distillate flux, distillate purity and temperature polarization. According to their results, the energy efficiency of MD can be improved by increasing the driving force on the membrane. They found MD as a potential treatment method for textile effluent and the recovery of quality water for re-use. Table 6 shows some other applications of MD in treatment of wastewater.

Characteristically, membranes for MD should have low resistance to mass transfer in order to enable free flow of mass. To enhance heat maintenance in the system, membrane material must have a low thermal conductivity and very importantly also, a membrane for MD must also have low affinity for water to guard against unnecessary wetting of the membrane. Pore sizes usually range from 0.1 µm to 1 µm [98]. MD has many attractive prospects. It can be driven by renewable energy sources such as solar or wind. Waste heat recovered from industrial processes can also be used. In terms of pressure requirements, MD uses low hydrostatic pressure compared to Reverse Osmosis (RO). For example, MD can be performed at operating pressures near atmospheric pressure. Again, as compared to pressure driven membrane processes, membrane fouling is less due to larger pore size requirements. In terms of separating nonvolatile materials from volatile ones, feed product separation is 100%. Contaminant concentration has no influence on product quality [107,108].

All these notwithstanding, MD has some drawbacks. Firstly, poor history of its usage, leads to uncertain water production costs (WPC). Secondly, non-availability of membranes specifically designed for MD puts the process at very high risk when membranes meant for other processes are employed. This can lead to membrane wetting, which in turn creates room for organic deposits and consequently leading to vigorous pretreatment requirements. This makes the process more expensive. Finally, since heat and mass transfer take place simultaneously, a fluid boundary layer is formed, which leads to temperature polarization (TP). TP is a phenomenon that occurs as a result of difference in temperature between the bulk of the feed and the feed-membrane interface (where evaporation of water occurs) and the difference in temperature between the permeate-membrane interface (where condensation occurs) and the permeate itself. This temperature polarization has a negative effect on the driving force, leading to lower permeate flux [7,52,98,109].

#### 3.3.1. Membrane Distillation Configurations

**Figure 7:**
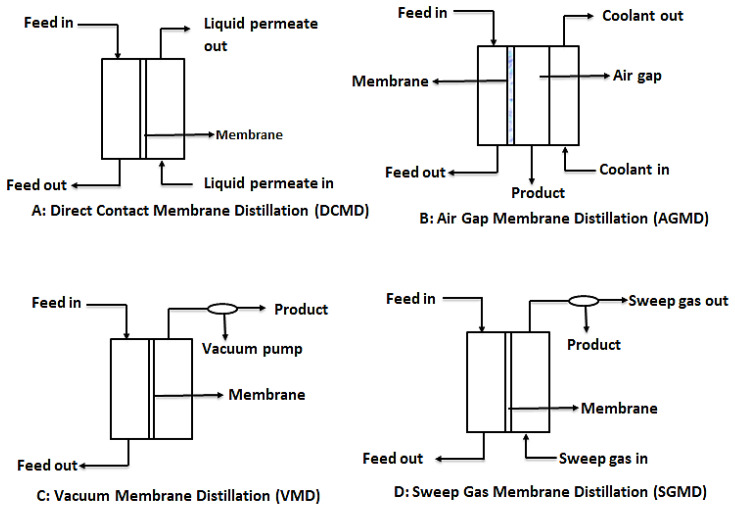
Schematic diagrams for the different types of MD configurations. Modified from [110].

In order to provide the needed driving force for MD, four main configurations are usually considered as shown in Figure 7. These are: direct contact membrane distillation (DCMD), air gap membrane distillation (AGMD), vacuum membrane distillation (VMG), and sweeping gas membrane distillation (SGMD) [110]. However, recent developments in MD have considered hybrid combinations such as thermostatic sweeping gas membrane distillation (TSGMD) and liquid gap membrane distillation (LGMD) [111]. These configurations are briefly discussed below.

#### 3.3.2. Direct Contact Membrane Distillation (DCMD)

DCMD is the most used MD configuration. In this process, like conventional distillation, the hydrophobic microporous membrane maintains a direct contact between the feed and the permeate. This happens because evaporation of water from the feed and condensation of this vapor occurs simultaneously, forming a liquid-vapor interface at the membrane pores. It is worthy to note that the vapor pressure difference is induced by the trans-membrane temperature difference, which is as a result of the lower temperature maintained at the permeate side of the membrane [7,110]. The main challenge with DCMD is the heat loss due to conduction.

#### 3.3.3. Air Gap Membrane Distillation (AGMD)

In AGMD, a stationary air gap is introduced between the membrane and the condensation surface. Vaporized molecules travel across the stationary air gap and condense on the cold surface within the membrane module by natural convection caused by the temperature difference in the air gap. While this module serves to reduce heat loss through conduction, the resultant permeate flux is low [112].

#### 3.3.4. Vacuum Membrane Distillation (VMD)

This design configuration makes use of vacuum, provided by a vacuum pump at the permeate side of the membrane. The constant low pressure provided by the vacuum pump must be lower than the saturation pressure of the volatile components of the feed. This low pressure enhances movement of the vaporized permeate. In effect, separation is achieved by the partial pressure difference across the membrane. Condensation of permeate takes place outside the membrane module [96].

#### 3.3.5. Sweeping Gas Membrane Distillation (SGMD)

As the name suggests, SGMD makes use of a gas, usually inert gas, to sweep away the vaporized permeate from the membrane permeate side. Since the condensation of the vaporized permeate occurs outside the membrane module, external condensers are needed to collect the product water. Even though this design prevents loss of heat through conduction due to the gas barrier created, the need for external condensers make it complicated [107,110].

#### 3.3.6. Thermostatic Sweeping Gas Membrane Distillation (TSGMD)

This is a modified form of SGMD and AGMD. In this design, a cold wall is added in the cold chamber. This cold wall helps minimize the increase in the sweeping gas temperature. Like the AGMD, some of the permeate condenses on the cold wall within the membrane module, whereas the rest is swept away by the inert gas into an external condenser, just like the SGMD [110,112,113].

#### 3.3.7. Liquid Gap Membrane Distillation (LGMD)

In this design, a third channel is introduced, on which the permeate condenses. This channel is usually a non-permeable foil which also separates the permeate from the coolant. This is very advantageous since it provides the option of choice of coolant. The foil also helps in reducing the overall temperature difference across the membrane [114,115]

## 4. Membrane Modules and Selection

For large scale membrane processes, like industrial or other commercial uses of membranes, large membrane areas are required. These large membrane areas are packaged economically into what is known as modules. There are mainly four types of membrane modules, namely plate and frame module, tubular module, spiral wound module and hollow fiber modules [13]. A summary of some of the properties of the membrane modules is shown in Table 7. These are further discussed briefly below.

### 4.1. Plate-and-Frame Module

**Figure 8:**
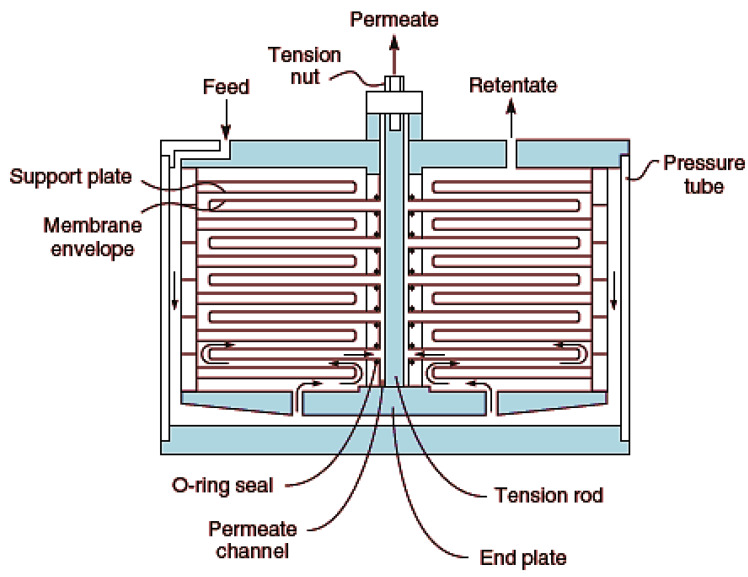
Plate and frame membrane module. Adapted from [13].

This is one of the earliest modules developed. It consists of the membrane, feed spacers and product spacers which are bedded together into a metallic frame [117]. These spacers prevent sticking together of the membrane as well as provide channels for flow of feed and product. It is worthy to note, however, that this module is not generally in use, but employed for special purposes like treatment of wastewaters with high levels of suspended solids, e.g., landfill leachate. Figure 8 shows a typical plate-and-frame design.

### 4.2. Tubular Module

This module consists of an outer housing, referred to as shell. This shell is tubular in nature. Within this tubular shell, is a perforated or porous stainless steel or fiberglass pipe, within which a semi-permeable membrane is embedded. The fluid to be treated is fed into the tube under pressure. The permeate from the membrane passes through the perforated pipe into the inside of the housing and then collected through the permeate outlet [118]. Tubular membranes are adapted to treating feed with high solid contents.

### 4.3. Spiral Wound Module

**Figure 9:**
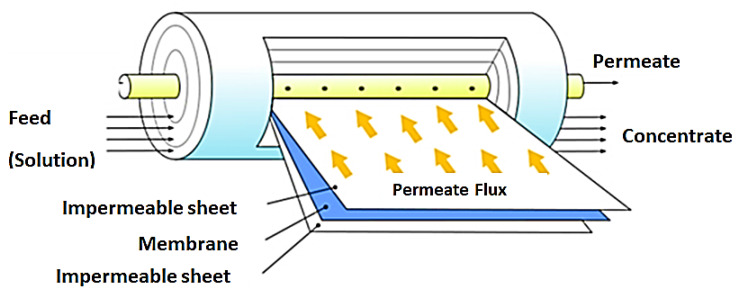
Spiral wound membrane module. Adapted from [121].

This membrane module is the most widely applied in RO and NF operations. The configuration offers a high packing density leading to high membrane surface area. This design is made up of a number of membranes, permeate spacers and feed spacers wound around a perforated central collection tube. These are in turn placed into a tubular pressure vessel. Water to be treated enters the spiral wound module at a tangent to the membrane. In this way, permeate flows perpendicular to the membrane surface, through the permeate spacers and finally collected in the central collection tube. [13,119]. This module has the advantage of easy replacement of module elements and scaled up for large scale operations [120]. Figure 9 shows a representation of this module.

### 4.4. Hollow Fiber Module

This module type houses a bundle of hollow fibers, whether closed or open end, in a pressure vessel. Hollow fibers consist of a porous nonselective support layer of about 200 µm and an active layer of thickness >40 nm. This active layer is the actual membrane, but needs support to be able to withstand the hydrostatic pressure [122].

**Figure 10:**
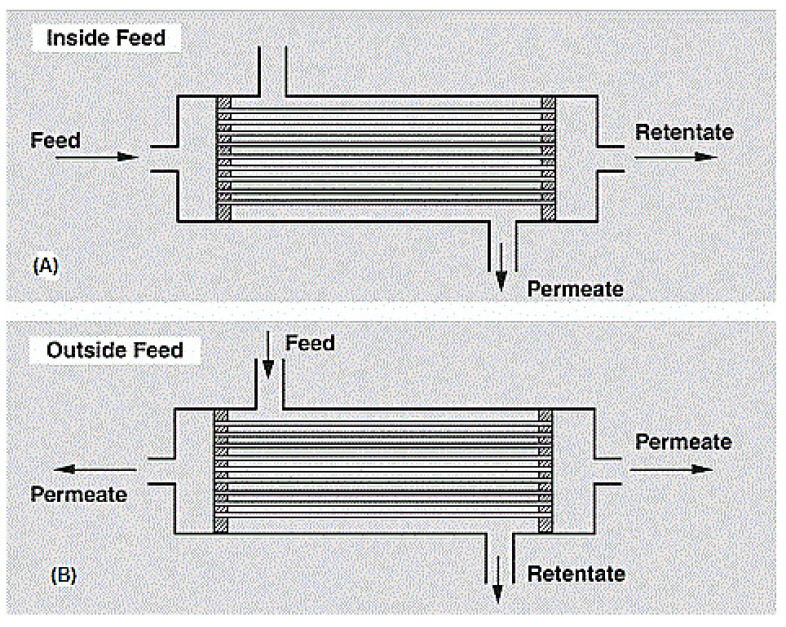
(**A**) Bore side feed hollow fiber membrane modules, (**B**) Shell side feed hollow fiber membrane module. Adapted from [122].

Hollow fiber modules are either shell-side (outside) feed types or bore-side (inside) feed types, depending on their use. For high pressure purpose applications (up to 70 bar), the shell-side feed type is preferred, whereas for low to medium pressure purpose applications, the bore-side feed type is preferred. Figure 10 shows a diagram of the hollow fiber membrane. A very notable advantage of this module type is its ability to house large membrane areas in a single module. However, it is very expensive to produce due to the sophistication of the production process and the huge capital requirements [13].

## 5. Concentration Polarization (CP)

CP is defined as a phenomenon where particle concentration near the membrane surface is higher than in the major part of the fluid [123]. CP is common to all membrane filtration processes. The mechanism of CP is such that a layer of accumulated solute particles is formed on the membrane surface as the permeate flows through the membrane. Since particle concentration is less in the permeate, there is a huge difference in concentrations of these particles at the permeate side and the feed side of the membrane [124]. Such a concentration difference would cause movement of solvent molecules backwards until equilibrium is formed. In FO, CP occurs within the porous support layer. This is known as internal concentration polarization (ICP). CP affects permeate flux, as the boundary layer formed as a result of accumulation of solute particles prevents easy movement of permeate through the membrane. Consequently, the longevity of the membrane is compromised. This eventually leads to high cost of the membrane process. Methods to reduce CP broadly fall under pretreatments, membrane modification, fluid management, or effective cleaning [125,126]. Pretreatment basically removes or reduces particles that contribute to concentration polarization. Section 6 discusses some pretreatment strategies used in membrane separation processes.

Membrane modification is mainly applied in FO membranes to deal with internal concentration polarization. In their study, Wang, et al. [127] developed a double skinned FO membrane using cellulose acetate (CA) which was found to be very promising in reducing ICP. Again, Chi, et al. [128] modified the surface of cellulose triacetate FO membrane (CTA) with magnetite nanomaterial to utilize the interforce between magnetic draw solutions and the magnetite to reduce ICP. The novel method was found to effectively reduce ICP in the FO membrane. In the same vein, Liu, et al. [129] modified a thin film composite (TFC) FO membrane surface with CaCO_3_ coated polyethersulfone which is highly hydrophobic. This improved the intrinsic ability of the membrane to draw water and resist ICP.

In pressure driven membrane processes, flow dynamics such as turbulence flow regimes, flow in curved channels, vibrations in membrane modules, pulsative flow techniques etc. are mainly used in controlling CP [125]. Mo, et al. [130] studied the effects of inserting spacers in a membrane channel on CP. The study showed that spacers can introduce some hydrodynamic conditions that reduce CP. In a review titled *Static Turbulent Promoters in Cross flow Filtration,* Bhattacharjee, et al. [131] noted that static mixers, kenics mixers, helical elements, cylindrical rods, thin wires, and spacers are widely utilized to induce turbulence in membrane filtration systems in order to control CP and enhance permeate flux. In an investigation conducted by Su, et al. [132], vibrations were imposed in RO membrane module during desalination to control CP. The authors found the technique to be useful in reducing CP and improving membrane flux. Periodic cleaning procedures, such as backwashing, back flushing, and chemical and physical cleaning, also play a good role in mitigating CP. They are discussed in more detail in Section 6.

## 6. Membrane Fouling and Pretreatment Strategies

Membrane fouling occurs when suspended solids, microbes, organic materials etc. are deposited on the membrane surface or within the membrane pores thereby causing decreased permeate flux [133]. Fouling can be considered irreversible when foulants (materials causing the fouling) are deposited in the pores of the membrane. When the foulants are merely deposited on the surface of the membrane, they form a cake layer which causes resistance to permeate movement. This fouling is considered reversible [133]. Membrane fouling affects the membrane performance as the movement of permeates is greatly hindered. Consequently, higher pressure than normal is needed to ensure passage of permeates through the membrane. The higher the fouling, the more the pressure required [116]. Membrane fouling has dire consequences on overall membrane performance. These include high energy consumption, more down time, reduction in membrane filtration area etc.

There are different forms of fouling, depending on the foulant. These include colloidal fouling, bio-fouling, organic fouling and inorganic fouling (scaling) [134]. Colloids can be either organic, inorganic, or as composites. These may include microorganisms, biological debris, polysaccharides, lipoproteins, clay, silt, oils, iron and manganese oxides etc. These materials accumulate and stick to membrane material over time [135]. Biofouling is the deposition and growth of biofilms on a membrane. These biofilms consist mainly of microbial cells and extracellular polymeric substances (EPS), which are formed as a result of the attachment of microorganisms to moist surfaces. In this medium, these organisms feed on accumulated nutrient in the system and grow, consequently blocking the pores of the membrane and increasing resistance to permeate flow [136].

Inorganic fouling (scale) is the deposition of inorganic salts on the membrane surface. These salts may include, but not limited to CaSO_4_, CaCO_3_, and SiO_2_ [137]. During the formation of the scales, poorly soluble salts precipitate out of solution onto the membrane surface when their concentrations exceed their solubility limits [138]. Organic fouling occurs when there is adsorption of organic compounds present in natural organic matter onto the surface of the membrane and accumulate over time, hindering permeate movement through the membrane [134].

It is worthy to note that membrane fouling depends on feed characteristics like pH and ionic strength, membrane characteristics like roughness, hydrophobicity etc., and process conditions like cross flow velocity, trans-membrane pressure and temperature. All these factors interact in one way or another to enhance membrane fouling [139,140].

### 6.1. Methods of Fouling Control: Membrane Cleaning

Membrane separation is largely a size exclusion mechanism. By principle, the particles rejected, end up fouling the membrane. This makes fouling in membranes inevitable. Some techniques have been proposed to reduce fouling in membranes. Relevance of these techniques depends on the properties of the feed solution and the membrane. Some of these techniques include: boundary layer velocity control, turbulence inducers, membrane material modification, and the use of external fields [141]. In the same vein, feed pretreatment, flow manipulation, rotating membranes, and gas sparging were recommended by Williams and Wakeman [142].

Membrane cleaning comes in to restore the permeation flux of a membrane which is lost as a result of fouling. This involves the removal of deposited materials on the membrane in order to pave way for movement of permeate. Membrane cleaning can be classified largely as physical, chemical, biological/biochemical or physico-chemical. Again, cleaning can be referred to as in-situ when the membrane module remains within the reactor during cleaning or ex-situ when the membrane module is removed and cleaned separately [143,144].

Physical cleaning: This involves mechanical treatment of the membrane to dislodge and remove foulants from the membrane [140]. These treatments include:

Periodic back flushing: this involves the application of pressure on the permeate side of the membrane, thereby causing backward movement of the permeate through the membrane. This causes deposited materials to be lifted off the membrane surface. The pressure applied to cause the backwash needs to be higher than the filtration pressure [140,142]. Backwashing is the most widely used fouling reversal technique used in industry. It is known to effectively regain flux from fouling caused by the deposition of materials on the surfaces of the membranes as a gel or cake layer. It is however not effective for removal of irreversible fouling, which is caused mainly by clogging of the membrane pores with colloidal suspensions and dissolved materials [145].

Pneumatic cleaning: This includes air sparging, air lifting and air scouring. Basically, this involves cleaning of the membrane with air under pressure. The air destabilizes and loosens the foulants from the membranes by causing a shear force on the membrane surface. Air may be used for direct cleaning or to enhance permeate flow by bubbling it through the feed. This process is advantageous for the fact that there is no chemical usage, however the cost of pumping air is a huge factor to contend with [143,146].

Ultra-sonic cleaning: This process utilizes ultrasound to cause agitation in a liquid medium. The formation, growth and collapse of bubbles during the process (cavitation) transmit energy in the form of turbulence to the membrane surface, which dislodges foulants from the membrane surface [147]. Since the waves are transmitted at the molecular level, ultrasonic cleaning is very effective in cleaning the surface of the membrane. This physical cleaning process depends on ultrasonic power, cleaning temperature, cross flow velocity and pulse duration [147,148,149]

Sponge ball cleaning: This involves the use of sponge balls to wipe the surface of membranes. The sponge ball, which is usually made of materials like polyurethane is inserted into the permeator and as it moves through the permeator, it scrubs the surface of the membrane, thereby scrapping off the foulants. This is a mechanical cleaning process which is applicable to tubular membranes with large diameters [140,150].

Chemical Cleaning is employed in situations with irreversible fouling. The basis of chemical cleaning is the knowledge of the interactions between foulant and membrane material, foulant and cleaning chemical and cleaning chemical and membrane material. These play a great role in selecting most appropriate chemical for the cleaning process [151]. Chemical cleaning is expected to have the following effects on a fouled membrane: loosen and dissolve foulant, keep foulant in solution, avoid causing new fouling, and not attack the membrane being cleaned. Chemical cleaning is done mainly as a cleaning in place (CIP) process, where the retentate channel is filled with the cleaning solution (detergent) which weakens the bonds of the foulant over time. This then gives room for normal cross flow to remove these foulants [152].

Generally, cleaning agents are classified as acids, alkalis/bases, chelating agents or sequestrants, enzymes, surfactants, or disinfectants. All these agents are accustomed to removing foulants of different composition or charge. For example, acid cleaning aims at removing inorganic foulants like salt precipitates or scales and metal oxides. The acids commonly used include hydrochloric acid (HCl) and sulphuric acid (H_2_SO_4_), nitric acid (NHO_3_) and phosphoric acid (H_3_PO_4_) [143,153]. Alkalis/bases are mainly employed at high pH levels (11–12) or less, depending on the nature of the membrane. They are used mostly in organic fouling cleaning. The main Alkalis/bases used is sodium hydroxide (NaOH). Other forms of alkalis employed include carbonates and phosphates [140,150].

Biological/biochemical cleaning is defined as the use of bioactive agents like enzymes, enzyme mixtures, or signal molecules for foulant removal from membranes [154]. Unlike physical and chemical cleaning that damage the membrane, biological and biochemical processes have low footprints in membrane cleaning and are more sustainable. In most cases, enzymatic cleaning, energy uncoupling and quorum quenching are applied cleaning operations especially in membrane bioreactors [144,154,155]. This type of cleaning is mostly employed in cleaning of membranes used in abattoir wastewater treatment.

Physico-chemical cleaning methods: As the name suggests, this method combines physical and chemical cleaning for foulant removal. This involves the addition of chemical agents to physical cleaning methods to enhance its effectiveness of the cleaning process. A typical example of physico chemical cleaning method is the chemically enhanced backwashing (CEB). Another example is the use of ultrasound in chemical cleaning which is able to enhance flux recovery of up to 95% [156,157].

### 6.2. Pretreatment Strategies for Membrane Processes

Pretreatment is the initial treatment given to wastewater prior to the application of membrane separation processes. Feed pretreatment plays an integral part in the success of membrane process. Pretreatments do not only reduce membrane fouling, they also contribute to energy utilization. Technically, pretreatments change the physical, chemical or biological properties of wastewater so as to make membrane separation more efficient [158].

Different methods are adopted as pretreatments to precondition influents for membrane separation. Physicochemical methods such as coagulation, adsorption and softening have been applied in several instances to pretreat wastewater before membrane separation [159]. In the treatment of produced water, Sardari, et al. [160] applied electrocoagulation as a pretreatment to DCMD. The results showed 57% water recoveries from produced water of containing 135 /L dissolved solids. In similar applications, Chang, et al. [161] and Kong, et al. [162] pretreated shale gas flow back water and produced water with chemical coagulation prior to treatment with UF. Both studies found a significant reduction in fouling of the membranes and maintenance of constant flux. These physicochemical pretreatment methods are efficient in removal of suspended solids and organic contaminants that have high membrane fouling abilities. There is also the combination of coagulation/flocculation and adsorption as pretreatment methods for membrane processes. This is to further enhance the removal of dissolved and colloidal substances from feed wastewater as proved by [163,164,165,166,167,168].

Pre-filtration is another method used as pretreatment to membrane processes. Pre-filtration may include the use of packed bed filters, strainers, filter cloths or low pressure membranes processes (some of which are shown in Table 2) [158]. In a pilot scale experiment, Tooker and Darby [169] pretreated secondary effluent from the wastewater treatment plant of the University of California, Davis using a cloth media filter for onward treatment by microfiltration. The final effluent was found to be of high quality, having low values of turbidity and BOD, as well as non-detectable levels of total and fecal coliform bacteria. In a similar application, López Zavala, et al. [170] employed felt and compressed polyester in the pretreatment of gray water from washing machine discharges. The pretreatment method was found to improve MF and UF performances in terms of an increase in flux and reduction in fouling rates. Again, in purification of olive mill (Peloponnisos, Greece) wastewater, Paraskeva, et al. [171] studied the combination of UF and RO. Prior to the UF, a polypropylene filter (80 µ) was used to pretreat the wastewater, removing suspended solids. The resultant effluent was fit for discharge and irrigation purposes.

Other forms of pretreatment include the use of dissolved air flotation [172,173] and biological pretreatment methods [174,175,176].

## 7. Recommendations for Further Research

Membrane technology is gradually revolutionalising water and wastewater treatment. Much work has been done in this area over the years. There is however still room for improvement in many areas. As fouling and high energy demand remain a major issue in non-equilibrium pressure driven processes, continuous research is needed to find a lasting solution to them, either through introduction of rigorous but cheap pre-treatment processes or through development of fouling resistant membranes. In membrane distillation, continuous studies are needed to adequately understand the concept of temperature polarization and, accordingly, developing suitable membranes will help make the process more viable for large scale application.

Improvements in draw solute recovery processes are needed to make FO applications cheaper. Future research should look at the possibility of other recovery methods for salt-based draw solutes. Further studies in ED, EDR, and pervaporation should focus more on membrane development and energy utilisation.

## 8. Conclusions

There is an unending list of membrane technology applications in wastewater treatment. This paper attempted to summarize the major ones that are used, citing examples of their application, their advantages and disadvantages, as well as some membrane related areas like fouling and module structures. Hopefully, this paper is useful in providing good information for further research into membrane technology applications in wastewater treatment.

## Figures and Tables

**Table 1:** Some features of pressure driven membranes. Adapted from [7,18].

Membrane Process	* MWCO (kilo Dalton)	Retained Diameters (µm)	Pressure Required (bar)	Membrane Type	Average Permeability (L/m^2^ h bar)	Solutes Retained
MF	100–500	10^−1^–10	1–3	Porous, asymmetric or symmetric	500	Bacteria, fat, oil, grease, colloids, organics, micro-particles
UF	20–150	10^−3^–1	2–5	Micro porous, asymmetric	150	Proteins, pigments, oils, sugar, organics, microplastics
NF	2–20	10^−3^–10^−2^	5–15	tight porous, asymmetric, thin film composite	10–20	Pigments, sulfates, divalent cations, divalent anions, lactose, sucrose, sodium chloride
RO	0.2–2	10^−4^–10^−3^	15–75	Semi porous, asymmetric, thin film composite	5–10	All contaminants including monovalent ions

* MWCO = Molecular weight cut off.

**Table 2:** Some applications of pressure driven membrane processes in wastewater treatment.

Pressure Driven Membrane Process	Wastewater Treated	Results *	Reference
UF	Vegetable oil factory	COD ^a^ (91%), TSS ^b^ (100%), TOC ^d^ (87%), PO_4_^3−^ (85%), Cl^−^ (40%)	[22]
MF-RO	Urban wastewater	Pesticides and pharmaceuticals removed to discharge limit	[23]
MF	Municipal wastewater (disinfection and phosphorus removal)	Contaminants removed to below detection limit	[24]
MF	Synthetic emulsified oily wastewater	95% removal of organic contaminants	[25]
NF-RO	Dumpsite leachate	95% water recovery	[26]
UF	Poultry slaughterhouse wastewater	COD and BOD ^c^ removal > 94%, fats (99%), suspended substances (98%)	[27]
NF	Textile	COD (57%), color (100%), salinity (30%)	[28]
UF-RO	Metal finishing industry	90–99% removal of different contaminants	[29]
UF-RO	Oily wastewater	Oil and grease (100%), TOC (98%), COD (98%), TDS ^e^ (95%), Turbidity (100%)	[30]
UF-NF/RO	Phenolic wastewater from paper mill	COD (95.5%), phenol (94.9%)	[31]

* Note: ^a^—chemical oxygen demand, ^b^—total suspended solids, ^c^—biochemical oxygen demand, ^d^—total organic carbon, ^e^—total dissolved solids.

**Table 3:** Applications of FO in wastewater treatment.

Application	Draw Solute Used	Result	Reference
Raw municipal wastewater	NaCl, MgCl_2_	Up to 70% water recovery	[40]
Coke-oven wastewater	NaCl, MgSO_2_ and CaCl_2_∙H_2_O (0.4–2.5 M)	96–98% removal of cyanide, phenols and COD	[35,41]
Reduction in volume of gas field produced water	1 M NaCl	50% of volume reduced	[42]
Coal mine wastewater desalination	More saline mine waster	More than 80% of volume of mine water recovered	[43]
Sewage (primary effluent)	NaCl, MgCl_2_∙6H_2_O	Low water recovery due to internal concentration polarization and fouling	[44]
Domestic wastewater	NaCl (35 g/L)	Over 90% contaminant removal	[45]

**Table 4:** Application of ED and EDR in wastewater treatment.

Application	Result	Reference
Treatment of almond industry wastewater	94% recovery of water	[61]
Treatment of university sewage	70-90% removal of TDS, total inorganic carbon, cations and anions. 23–52% removal of COD, BOD, colour, turbidity and TOC	[62]
Tertiary treatment of municipal wastewater	100% effectiveness in treatment to meet discharge standards and removal of Cl^−^, Mg^2+^, Ca^2+^	[63]
Treatment of drainage wastewater for agricultural purposes	Removal of heavy metals and Na^+^ up to 99%	[64]
Treatment of tannery wastewater	92–100% removal of COD, color, NH_3_-H, Cr.	[65]
Removal of heavy metals (* Cd and * Sn) from electroplating industry wastewater	Successful removal of Cd (74.8%) and Sn (64.5%)	[66]
Treatment of wastewater from the China Steel Corporation wastewater treatment plant	92% desalination rate, 98% Cl^−^ removal, 80% SO_4_ removal and 51% removal rate of COD	[60]

* Cd = Cadmium, * Sn = tin.

**Table 5:** Applications of pervaporation in the removal of specific contaminants.

Application	Results	Reference
Removal of toluene from aqueous solution	Up to 42% of toluene removed	[73]
1.0 mol% aqueous VOC (ethyl acetate, diethyl ether, acetonitrile)	Up to 90.35 * wt% removal	[74]
Removal of methyl tert-butyl-ether from aqueous solution	Up to 95% removal	[75]
Removal of 0.5 wt% pyridine from water	Effective removal reported	[76]
Removal of 0.39 wt% Isopropyl acetate from aqueous solution	Effective removal reported	[77]
Removal of 0.1–0.4 wt% phenol and chlorophenol from aqueous soloution	Effective separation reported	[78]

* wt% = Percentage by weight.

**Table 6:** Some application of MD in wastewater treatment.

Application	Results	Reference
Wastewater from nano-electronics industry	High quality permeate with contaminant separation efficiency of >99%	[102]
Stick water treatment using	Up to 78% water recovery and 99% salt rejection using * PU-PTFE commercial membranes	[103]
Treatment of RO retentate from flue gas desulphurization wastewater	87% water recovery	[104]
Dairy wastewater treatment	>99% rejection of Total organic carbons	[105]
Textile wastewater treatment	>99% dye rejection	[106]

* PU-PTFE = Polytetrafluorethylene with Polyurethane surface layer.

**Table 7:** Basic properties of various membrane modules (Adapted from [116]).

Property	Plate-and-Frame	Tubular	Spiral Wound	Hollow Fiber
Packing Density ft^2^/ft^3^ (m^2^/m^3^)	45–150 (148–492)	6–120 (20–374)	150–380 (492–1247)	150–1500 (492–4924)
Potential for fouling	Moderate	Low	High	Very High
Ease of Cleaning	Good	Excellent	Poor	Poor
Relative Manufacturing cost	High	High	Moderate	Low

## Abbreviations

AEM	Anion exchange membrane
AGMD	Air gap membrane distillation
BOD	Biochemical oxygen demand
CA	Cellulose acetate
Cd	Cadmium
CEB	Chemically enhanced backwashing
CEM	Cation exchange membrane
CIP	Cleaning in place
COD	Chemical oxygen demand
CP	Concentration polarization
DCMD	Direct contact membrane distillation
ED	Electrodialysis
EDR	Electrodialysis reversal
FO	Forward osmosis
FO-RO	Forward osmosis-reverse osmosis
ICP	Internal polarization
LGMD	Liquid gap membrane distillation
MD	Membrane distillation
MF	Microfiltration
MWCO	Molecular weight cut off
NF	Nanofiltration
PE	Polyethylene
PTFE	Polytetraflourethylene
PV	Pervaporation
RO	Reverse osmosis
TDS	Total dissolved solids
TFC	Thin film composite
TOC	Total organic carbon
TSGMD	Thermostatic sweeping gas membrane distillation
TSS	Total suspended solids
SGMD	Sweeping gas membrane distillation
Sn	Tin
UF	Ultrafiltration
VMD	Vacuum membrane distillation
